# High Potential Source for Biomass Degradation Enzyme Discovery and Environmental Aspects Revealed through Metagenomics of Indian Buffalo Rumen

**DOI:** 10.1155/2014/267189

**Published:** 2014-07-17

**Authors:** K. M. Singh, Bhaskar Reddy, Dishita Patel, A. K. Patel, Nidhi Parmar, Anand Patel, J. B. Patel, C. G. Joshi

**Affiliations:** ^1^Department of Animal Biotechnology, College of Veterinary Science and Animal Husbandry, Anand Agricultural University, Anand, Gujarat 388001, India; ^2^Xcelris Genomics Xcelris Labs Ltd, Old Premchandnagar Road, Opp. Satyagrah Chhavani, Bodakdev, Ahmedabad 380054, India; ^3^Department of Animal Nutrition, Livestock Research Station, Sardarkrushinagar Dantiwada Agricultural University, Gujarat 385506, India

## Abstract

The complex microbiomes of the rumen functions as an effective system for plant cell wall degradation, and biomass utilization provide genetic resource for degrading microbial enzymes that could be used in the production of biofuel. Therefore the buffalo rumen microbiota was surveyed using shot gun sequencing. This metagenomic sequencing generated 3.9 GB of sequences and data were assembled into 137270 contiguous sequences (contigs). We identified potential 2614 contigs encoding biomass degrading enzymes including glycoside hydrolases (GH: 1943 contigs), carbohydrate binding module (CBM: 23 contigs), glycosyl transferase (GT: 373 contigs), carbohydrate esterases (CE: 259 contigs), and polysaccharide lyases (PE: 16 contigs). The hierarchical clustering of buffalo metagenomes demonstrated the similarities and dissimilarity in microbial community structures and functional capacity. This demonstrates that buffalo rumen microbiome was considerably enriched in functional genes involved in polysaccharide degradation with great prospects to obtain new molecules that may be applied in the biofuel industry.

## 1. Introduction

Livestock production in India is subsidiary to plant production. In tropical countries, the ruminants are fed on lignocellulosic agricultural byproducts. Ruminants digest such plant materials by virtue of the extensive microbial community [1, 2], which are found in the rumen and provide the host with nutrients, predominantly in the form of volatile fatty acids and microbial protein [3]. The rumen habitat contains a consortium of microorganisms that harbour the complex lignocellulosic degradation system for the microbial attachment and digestion of plant biomass. However, the complex chemical processes required to break down the plant cell wall are rarely carried out by a single species. Evidence also suggests that the most important organisms and gene sets involved in the most efficient hydrolysis of plant cell wall are associated with the fiber portion of the rumen digesta [4]. Plant cell walls have a basic structure of cellulose surrounded by a complex matrix of hemicellulose, pectin and protein, cell types, and stages of maturity [5].* Ruminococcus flavefaciens, Ruminococcus albus,* and* Fibrobacter succinogenes* are considered to be the most important cellulose-degrading bacteria in the rumen [6], and they produce a set of cellulolytic enzymes, including endoglucanases, exoglucanases, and glucosidases, as well as hemicellulases. In addition, the predominant ruminal hemicellulose-digesting bacteria such as* Butyrivibrio fibrisolvens* and* Prevotella ruminicola* degrade xylan and pectin and utilize the degraded soluble sugars as substrates [7]. In recent years, rumen metagenomics studies have revealed the vast diversity of fibrolytic enzymes, multiple domain proteins, and the complexity of microbial composition in the ecosystem [8, 9].

The glycoside hydrolases (GHs) are modular enzymes that hydrolyse glycosidic bonds of carbohydrates, with classification based on amino acid sequence and predicted three-dimensional structure. Such enzymes may contain single or multiple catalytic modules (GH) together with single or multiple noncatalytic carbohydrate-binding modules (CBMs) [10]. Conversely, hitting upon the polysaccharide degrading enzyme machineries from metagenomic data is constraint [11, 12]. The microbes present in the rumen are not all culturable; moreover, if cloning is opted, enormous screening of clones will be required for covering the entire metagenome [12, 13]. However, there are limitations to metagenome mining [14], and the number of clones needed to represent the entire metagenome is staggering [15]. It has been reported that the nature of diet is one of the factors that shapes the composition of the gut microbiota [16]. Therefore, in order to improve the digestibility, the modulations of microbial consortia have also been attempted by dietary interventions [17].

Next-generation sequencing technologies have been used to characterize the microbial diversity and functional capacity of a range of microbial communities in the gastrointestinal tracts of humans [18, 19] as well as in several animal species [20–24]. Several groups have succeeded practice in metagenomic gene discovery of biomass-degrading genes from cow rumen and termite gut [9, 25].

The bovine rumen provides a unique genetic resource for the discovery of plant cell wall-degrading microbial enzymes (CAZymes) for use in biofuel production, presumably because of coevolution of microbes and plant cell wall types [9]. Identification of potent cellulolytic and other carbohydrate-active enzymes is of great interest for industrial applications [13]. Shotgun sequencing of the buffalo rumen metagenome was conducted to identify taxonomic diversity, metabolic makeup and discovers putative carbohydrate-active genes in the consortia.

## 2. Materials and Methods

### 2.1. Experimental Design and Rumen Sampling

The experimental animals were maintained for feeding experiments at Livestock Research Station, Sardarkrushinagar Dantiwada Agricultural University, Gujarat. Experiment was performed with the approval of the Anand Agricultural University, Institutional Animal Ethics Committee (Permission letter: AAU/GVC/CPCSEA-IAEC/108/2013). Eight 4- to 5-year-old healthy Mehsani breed of water buffaloes (*Bubalus bubalis*) were assigned to two basal diets groups (*n* = 4) based on green and dry roughages. The experimental diets were designed to have an increasing concentration of dry roughage and a decreasing concentration of the concentrate mix. The diets (dry roughage: concentrate and green roughage: concentrate) were M1D (50% dry roughage: 50% concentrate), M2D (75% dry roughage: 25% concentrate), and M3D (100% dry roughage); M1G (50% green roughage: 50% concentrate) and M2G (75% green roughage: 25% concentrate); M3G (100% green roughage).The experimental animals received M1 diet for six weeks followed by M2 for six weeks and then M3 for subsequent six weeks. The animals were maintained on each diet for six weeks to allow for microbial adhesion and adaptation to the new diet. On the last day of each experimental feeding period, rumen samples were collected 3 h after feeding using stomach tube. Each rumen sample was further separated to solid and liquid fractions by squeezing through a four-layered muslin cloth. Samples were immediately placed on ice, transported to the laboratory, and then stored at −80°C prior to metagenome analyses.

### 2.2. DNA Extraction

For isolation of DNA from liquid samples, the samples were thawed at room temperature and were then centrifuged at 5000 rpm for 5 min. The supernatant obtained thereafter was subjected to DNA isolation using commercially available QIAmp DNA stool mini kit (Qiagen, USA). For DNA extraction from solid samples, the samples were resuspended in phosphate buffer saline and vortexed for one and half hours for dislodging the tightly adhered bacteria from the solid feed particles. The samples were then centrifuged and the supernatant was subjected to DNA isolation using the same kit which was used for liquid sample. DNA samples were measured on a Nanodrop ND-1000 spectrophotometer (Thermo Scientific) to assess DNA quantity.

### 2.3. Ion Torrent Shotgun Sequencing

The shot gun sequencing on Ion Torrent PGM was performed at the Department of Animal biotechnology, College of Veterinary Science and Animal Husbandry, Anand Agricultural University, Anand, Gujarat, India. In brief, libraries were generated using the Ion Xpress plus fragment library kit (Life Technologies). The quality and quantity of generated libraries was assessed using the Agilent Bioanalyzer (Agilent Technologies) with Agilent High Sensitivity DNA Kit (Agilent Technologies), again quantified with Qubit fluorometer (Life Technologies). Quality check passed libraries were subjected to emulsion PCR using the Ion PGM 200 Xpress Template Kit (Life Technologies). After bead enrichment, beads were loaded onto Ion 316 chips and sequenced using an Ion Torrent PGM.

The data were then analyzed on Metagenome Rapid Annotation using Subsystem Technology (MG-RAST) server. The reads which passed the MG-RAST Quality filters were subjected to M5NR database (M5 nonredundant protein database, http://tools.metagenomics.anl.gov/m5nr/) for functional and diversity analysis. The 5 M's in M5 stand for the intersection of “Metagenomics, Metadata, Meta-analysis, Models, and Meta infrastructure” which target to synthesize the multiple databases with a unified standard and annotation of the metagenomic data in a more comprehensive and effective manner. The M5NR is a single searchable novel nonredundant database containing protein sequences and annotations from multiple sources and associated tools. Furthermore, the functional hierarchical classification was illustrated by using SEED subsystem. The sequences were compared using the BLASTX algorithm with an expected cut off of 1 × 10^−5^ [26].

### 2.4. Bioinformatics Analysis

The data analyses were performed with Prinseq and Metagenome Rapid Annotation using Subsystem Technology (MG-RAST) pipelines. Quality filter reads were subjected to M5NR database for functional and diversity analysis. The M5NR is a single searchable novel nonredundant database containing protein sequences and annotations from multiple sources and associated tools. The functional annotation and classification relied on the SEED subsystem [27]. The maximum *E*-value of 1*e* − 5, minimum percent identity of 60, and minimum alignment length of 50 pb were applied as the parameter settings in the analysis. Hierarchal clustering was performed using Ward's minimum variance with unscaled Bay Curtis distances (MGRAST).

Annotations based on the carbohydrate-active enzymes database [10] (http://www.cazy.org/) were performed for all the reads that passed the MG-RAST QC filter at an *E* value restriction of 1 × 10^−6^. Contigs sequences of the metagenomes were screened against PfamA database [28] by Pfam_scan [29] for particular glycoside hydrolase (GH) families and carbohydrate-binding module (CBM). The results were analyzed manually for proportion of different CAZymes. The profile of CAZymes of Buffalo rumen was also compared with cow http://ftp-trace.ncbi.nlm.nih.gov/sra/sra-instant/reads/ByRun/sra/SRR/SRR094/SRR094418/ and termite hindgut (downloaded from: ftp://ftp.metagenomics.anl.gov/projects/28/4442701.3/raw/2624.fna.gz). All the contigs from cow and termite metagenomes were further processed same as our data and uploaded in CAZy with the same parameters. 

## 3. Results and Discussion

The analysis of the reads yielded a high percentage of species identification in complex and dynamics metagenomes and even higher in less complex samples. Sequence reads from Ion Torrent provided enough specificity that is needed to compare the sequenced reads with the suitable databases and allowed the unambiguous assignment of closely related species. The shot gun sequencing runs of all metagenomics samples together yielded 3914.94 MB data. Prior to further processing, the raw read data were subjected to the MG-RAST online server [27] to remove duplicate and low quality reads. The unique sequence reads that passed the QC filtering step were then subjected to further analysis of taxonomic and functional annotation. The summary of metagenome data is presented in Table 1. In present study, metagenomic sequences were used to characterize genetic and functional capability of rumen microbiota of the buffalo.

### 3.1. Metabolic Profiles of the Buffalo Rumen Metagenome

Carbohydrate metabolism is the second most abundant functional category, representing 11.45–13.0% of the buffalo rumen metagenomes (Supplementary Table 1; Supplementary Material available online at http://dx.doi.org/10.1155/2014/267189). Genes associated with amino acid and derivatives, protein metabolism, cofactors (vitamins, prosthetic groups, and pigments), membrane transport, cell wall, and capsule. RNA metabolism and DNA metabolism are also abundant in the cow rumen metagenomes [8] as well as in Surti buffalo rumen [30]. Approximately 15.92–16.97% of the annotated reads from the buffalo rumen metagenomes were categorized within the clustering-based subsystems, most of which have unknown or putative functions. Metabolism-based hierarchical clustering demonstrates that all the buffalo rumen metagenome clustered together. All the samples were similar/dissimilar to each sample to the buffalo rumen (Supplementary Figure 1). The similarity/dissimilarty of function among all buffalo rumen is not surprising, considering the fact that they are all with similar digestive tract structures and functions.

### 3.2. Uncovering CAZymes Form Buffalo Rumen Metagenomes

**Figure 1:**
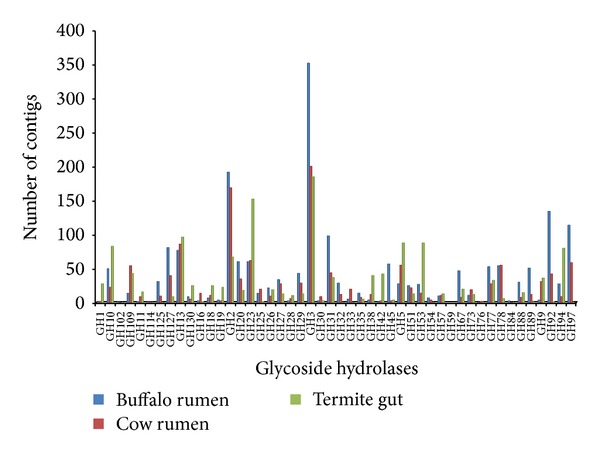
Comparison of predicted carbohydrate-active genes glycoside hydrolase in three cellulosic metagenomes: cow rumen microbiome, termite gut microbiome, and buffalo rumen microbiome.

**Figure 2:**
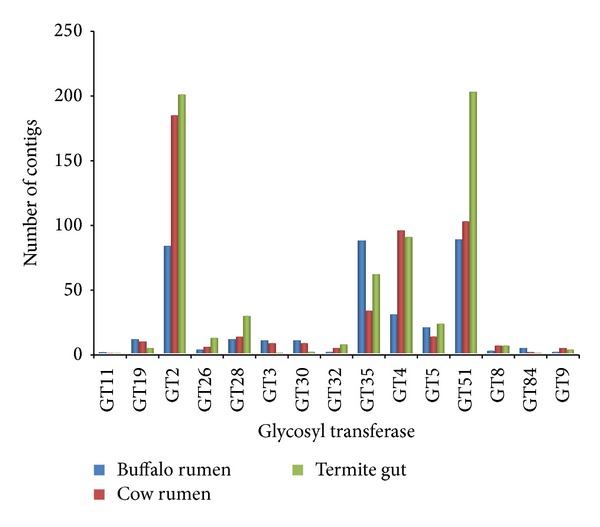
Comparison of predicted carbohydrate-active genes glycosyl transferase (GT) in three cellulosic metagenomes: cow rumen microbiome, termite gut microbiome, and buffalo rumen microbiome.

Rumen fluid is an excellent sample for mining CAZymes due to its apparent selection for evolution as a complex lignocellulosic degradation system [8]. We subjected total contigs to the carbohydrate-active enzymes database (CAZy; http://www.cazy.org), as described by Cantarel et al. [10], to obtain a more in-depth view of the carbohydrate enzymes present. The comparison of the all metagenome reads post-QC processing based on the CAZy database provided 2614 hits at an *E* value restriction of 1 × 10^5^. Candidate sequences that belong to the glycoside hydrolase GH3 (353) and families GH2 (192), GH92 (135), and GH97 (135) are the most abundant, followed by members of the glycosyl transferase families GT51 (89), families GT35 (88), and families GT2 (84) (Figures 1 and 2). Many genes encoding cellulase have been reported in buffalo rumen [31].

**Figure 3:**
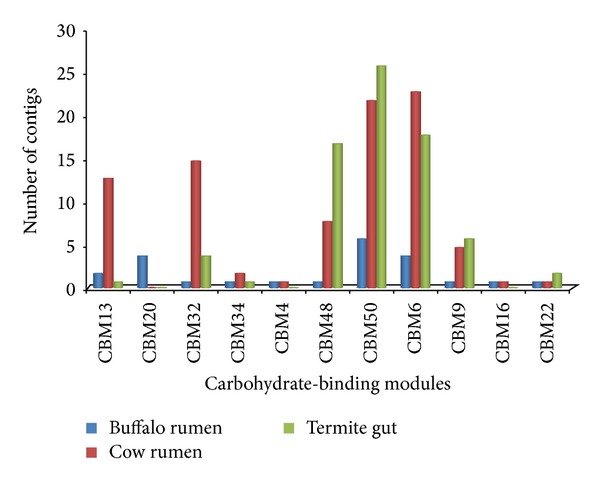
Comparison of predicted carbohydrate-active carbohydrate-binding modules (CBM), in three cellulosic metagenomes: cow rumen microbiome, termite gut microbiome, and buffalo rumen microbiome.

**Figure 4:**
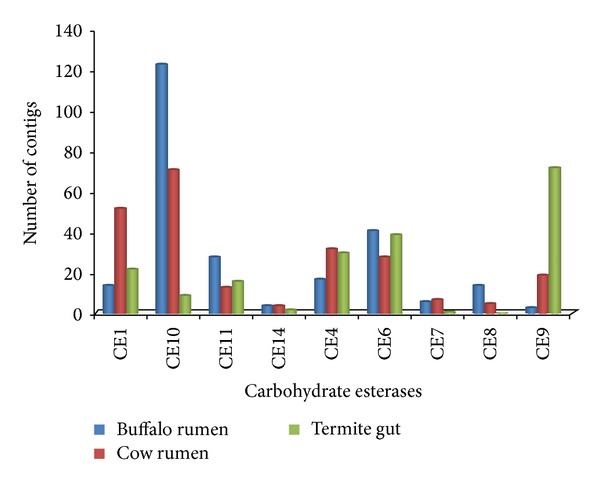
Comparison of predicted carbohydrate-active genes carbohydrate esterases in three cellulosic metagenomes: cow rumen microbiome, termite gut microbiome, and buffalo rumen microbiome.

**Figure 5:**
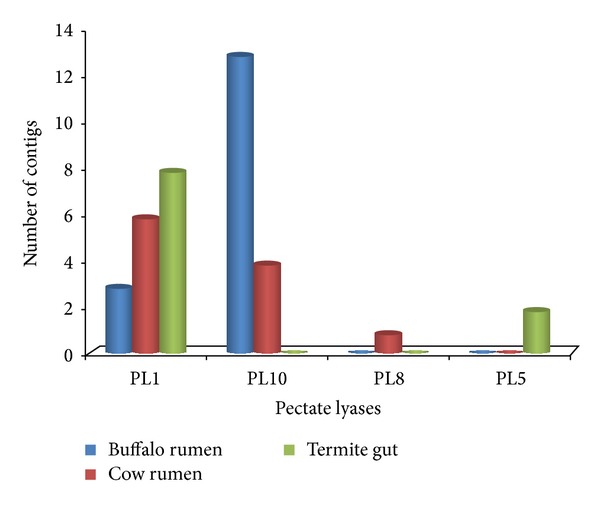
Comparison of predicted carbohydrate-active genes pectate lyases (PL) in three cellulosic metagenomes: cow rumen microbiome, termite gut microbiome, and buffalo rumen microbiome.

In the category of CBM, sequences that belong to the CE10 (124) are the most abundant (Figures 3 and 4). In addition sequences assigned to family PL are very scanty (Figure 5). Novel carbohydrate-binding module have been identified in a ruminal metagenome [32]. Carbohydrate-binding modules which are one of the structural components of cellulosomes-free enzyme system involved in carbohydrate digestion were also found. Though they were scantly represented (only 19 contigs) their occurrence is in accordance with the finding that microbiome of ruminants render cellulolytic bacteria associated with cellulosome complexes [2, 33].

GHs are a prominent group of enzymes that hydrolyze the glycosidic bond among the carbohydrate molecules. It is interesting to notice that there is a wide diversity of GH catalytic modules in the buffalo rumen microbiome, indicated by the 1943 modules belonging to 48 GH families. The most frequently occurring GH families in the buffalo rumen metagenome were GH3, GH2, and GH92 (Figure 1). Large-scale metagenomic sequencing of hindgut bacteria of a wood-feeding higher termite revealed that GHF5 was predominant in all identified GH families [25]. The most common activities of GH3 include b-D-glucosidases, a-L-arabinofuranosidases, b-D-xylopyranosidases, and N-acetyl-b-D-glucosaminidases [34]. In several cases, the enzymes have dual or broad substrate specificities with respect to monosaccharide residue, linkage position, and chain length of the substrate, such as a-L-arabinofuranosidase and b-D-xylopyranosidase [35]. GH2 components are b-D-galactosidases, b-glucuronidases, b-D-mannosidases, and exo-b-glucosaminidase. GH43 shows b-xylosidase, b-1, 3-xylosidase, a-L-arabinofuranosidase, arabinanase, xylanase, and galactan 1, 3-b-galactosidase activity (http://www.cazy.org/). Recently, Bashir et al. [36] studied the diversity of microbes existing in the guts of arthropods and their roles in biomass degradation and identified 42 unique cellulase-producing microbial strains and major glycosyl hydrolase enzymes.

Many candidate genes that were identified in buffalo rumen metagenome that belong to the glycosyl transferase families GT51, GT2, and GT35 are the most abundant (Figure 2). Glycosyl transferases are ubiquitous enzymes that catalyze the attachment of sugars to a glycone [37]. Amongst hemicellulases, 4 families were found (GHs 10, 11, 26, and 28) which jointly represented a total of 4% of all GHs. Pectin degrading pectin lyases (PL1, 10) was also obtained. Besides, we also identified xylan esterases (CE 1, 4, 6 and 7) and pectin methyl esterase (CE8), which acts on side chains of hemicellulose and pectin, respectively, and render both the large molecules accessible for further breakdown (Figures 4 and 5).

**Figure 6:**
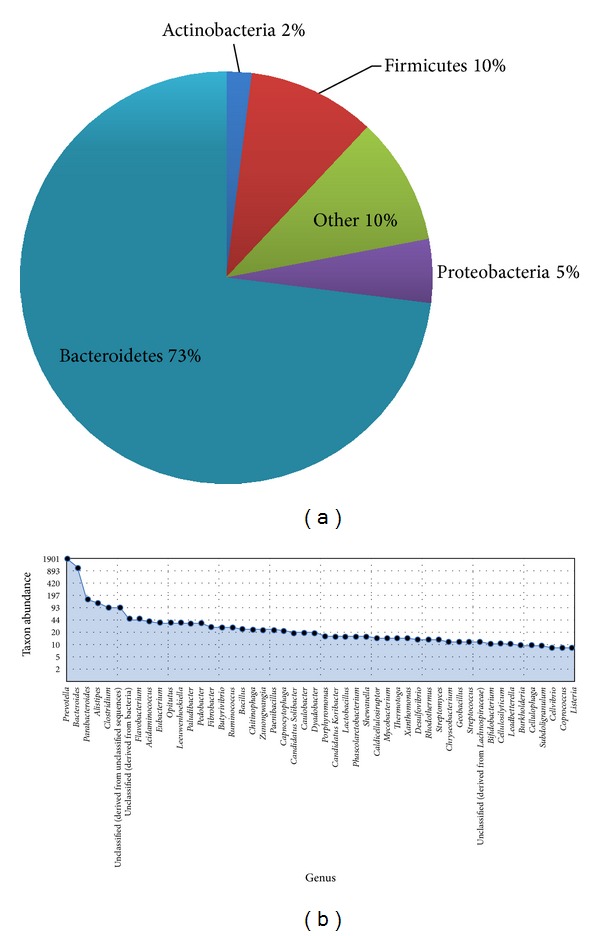
Taxonomic classification of putative CAZymes contigs and their microbial origin. (a) The pie chart shows the abundance of phylum and (b) genus abundances ordered from the most abundant to least abundant.

Metagenomes of termite hindgut [25] and cow rumen [9] were chosen for comparison with our data. Analysis indicates that buffalo metagenome had the highest amount of debranching enzymes (5.34%) and oligosaccharide degrading enzymes (25.2%) in which GH3 was predominant accounting for about 18.3% (Table 2). The higher proportion of oligosaccharide degrading enzymes naturally results into rapid formation of simple sugars which means that there will be faster and higher production of VFA (volatile fatty acid). However, the proportion of cellulases (8.91%) and hemicellulases (9.36%) in particular proportion of GH5 (5.71%) and GH10 (5.39%), respectively, was highest in termite. In addition, the proportion of oligosaccharide degrading enzymes GH32 and GH42 was also found to be highest in termite gut metagenome. Since wood has a greater proportion of cellulose and hemicellulose than the forage, the higher proportion of the GH5 and GH10 families in termite hindgut may be ascribed to its feed type. Similar to other metagenomes, buffalo rumen metagenome was also found lacking in enzyme families like GH6, 7, 48, 12, and 62. Furthermore, the contigs showing hits for CAZymes were analyzed to know the taxonomic placement [27]. Phylum Bacteroidetes represented highest percentage of contigs (73.0%) flowed by Firmicutes, Proteobacteria, and Actinobacteria (Figure 6(a)). Among Bacteroidetes, polysaccharide-degrading* Prevotella* genus was most abundant in the rumen of buffalo (Figure 6(b)). Bacteroidetes has been well reported for their starch, pectin, and xylan digestion [38].

### 3.3. Taxonomic Analysis of Buffalo Rumen Microbiota

The taxonomic computations provided 89.6–97.6% bacteria, 1.4–9.1% eukaryota, 0.8–1.8% Archea, and 0.20–0.37% viruses (Table 3). In the question metagenomes, Bacteroidetes was the most predominant phylum (30–60%), followed by Firmicutes (20–40%), Proteobacteria (8–10%), and Actinobacteria (3–5%) in all samples (Supplementary Figure 2). This finding is consistent with a previous study [3] in cow rumen metagenome and they characterized the rumen bacterial populations of 16 individual lactating cows and showed 51% similarity in bacterial taxa (Firmicutes, Bacteroidetes, Proteobacteria, and Actinobacteria) across samples. In addition they also identified 32 genera that are shared by all samples, exhibiting high variability in abundance across samples. Jami et al. [39] have also reported predominance of Bacteroidetes, Firmicutes, and Proteobacteria (core microbiota) in lactating cow fed 30% roughage and 70% concentrate. Compared with our previous 16S rRNA gene based data [40], higher percentages of Firmicutes and lower percentages of Bacteroidetes in the Indian Surti buffalo fed green fodder Napier bajra 21 (*Pennisetum purpureum*), mature pasture grass (*Dichanthium annulatum*), and concentrate mixture (20% crude protein, 65% total digestible nutrients) rumen metagenome were observed. These differences may have been caused by the biases associated with the primers, PCR reaction conditions, or selection of clones [41].

Among the Bacteroidetes group, Bacteroidales were the most predominant, among which genera* Prevotella* and* Bacteroides* were consistently overrepresented (Supplementary Figure 3). The genus* Prevotella* was highly represented in shared microbial community; it was the most abundant bacterial genus in buffalo rumen metagenomes. This finding is consistent with a previous study by Li et al. [42] in which several bacterial species were quantified in ruminal samples. The study reported the predominance of* Prevotella* members, which comprised 42 to 60% of the bacterial rRNA gene copies in the samples [38].

Firmicutes were the second predominant phylum in the buffalo rumen microbiota with Bacilli and Clostridia as the primary contributor to the Firmicutes populations. The major genus in the Firmicutes phylum is* Clostridium*(Supplementary Figure 3). However, the fiber degrading bacteria,* Ruminococcus albus* and* Ruminococcus flavefaciens,* are less abundant, which is contrast to our previous study [43] in Surti buffalo rumen. Surprisingly, some bacterial taxa were less abundant from the core groups identified by shotgun sequencing and considered crucial for fiber degradation in the rumen. Notably the phylum Fibrobacteres, which includes one of the main cellulolytic bacteria,* Fibrobacter succinogenes* which is of great importance for rumen function, was found in only one-third of the samples. Several studies of the rumen microbiome have suggested that the abundance of this phylum and in particular* F. succinogenes* varies considerably across ruminant and diets. This was evident in a recent metagenomic study in which this phylum was completely absent from the fiber-adherent and total overall rumen microbiome [8].

Phylogenetic level and the metabolic level clustering of forty-eight individual metagenomes were carried out with unscaled Bay Curtis variance distances and presented through a double hierarchical dendrogram (Supplementary Figure 4). In the phylogenetic comparison, the Bacteroidetes, Firmicutes, Proteobacteria, and Actinobacteria were the most abundant with different proportions in all the metagenomes. These phyla are recognized to be omnipresent and dominant in the rumen [8, 26, 44]. The results are corresponding with previous studies, indicating that microbial community composition is similar across animals [3]. The microorganism composition of the animal gastrointestinal tract reflects the constant coevolution of the animal with its host [9]. The bacterial taxa may vary considerably between buffalo rumen; they appear to be phylogenetically related. This suggests that the functional requirement imposed by the rumen ecological niche selects taxa that potentially share similar genetic features. The heat map also demonstrates that the buffalo rumen metagenome contains lower Fibrobacteres, an important phylum of cellulose-degrading bacteria.

In the present study, we have identified cellulose and hemicelluloses encoding contigs from microbial community in buffalo rumen. Cellulose and hemicellulose are the major components of plant cell walls and the most abundant biopolymeric materials [45]. The natural breakdown of plant matter performed by hemicellulases has been exploited by biotechnologists to produce bioethanol [46]. Rubin [44] has identified and characterized 4 highly active beta-glucosidases from fibre-adherent microbial community from the cow rumen. All enzymes were most active at temperatures 45–55°C and exhibited high affinity and activity towards synthetic substrate and natural cello-oligosaccharides. They suggest that beta-glucosidases (animal digestomes) may be of a potential interest for bioethanol production in combination with low dosage of commercial cellulases.

## 4. Conclusion

The work presented here describes the composition of the overall functional capacity related carbohydrates genes and taxonomic communities of the buffalo rumen ecosystem. Major carbohydrates utilizing genes covering GH, CBM, and GT families were detected in abundance. In addition, results revealed that GH 3 was the most dominant among all the detected GH families. The high magnitude of glycosyl transferase and carbohydrate esterases suggests the development of combined action on biomass degradation process. In the present study four phyla dominated in microbiomes, namely, Bacteroidetes, Firmicutes, Proteobacteria, and Actinobacteria. The information obtained in this research will open new horizons towards a full understanding of the functional genes and metabolic capabilities of the biomass degrading microorganisms, with great prospects to obtain new molecules that may be applied in the biofuel and agricultural industry. In addition, the contigs generated from the buffalo rumen metagenome represent the vital information for isolating the potential enzymes for biofuel and other industrial applications.

## Supplementary Material

Table S1: Assignment of Gene Fragments to Functional Groups.
Table S2: Gene Assignment to Taxonomic group (48 Metagenomes).
Suppl Figure1: Metabolic clustering of 48 buffalo rumen metagenomes. A double hierarchical dendrogram was established through weight-pair group clustering methods based on the non-scaling Bay Curtis distance. The dendrogram shows the functional categories of the 48 metagenomes. The linkages of the dendrogram are based on the relative abundance of metabolic profiles. The heat map depicts the relative percentage of each phylum of microorganisms in each sample. The heat map color represents the relative percentage of the microbial descriptions in each sample.
Suppl Figure 2: A comparative abundance of organism with variable roughage diet respect to 50%, 75% and 100% GR and DR in GL, DL, GS and DS phase at Phyla level. (GR: Green roughage, DR: Dry roughage, GL: Green Liquid, DL: Dry Liquid, GS: Green Liquid and DS: Dry solid)
Suppl Figure 3: A comparative abundance of organism with variable roughage diet respect to 50%, 75% and 100% GR and DR in GL, DL, GS and DS phase at Genus level. (GR: Green roughage, DR: Dry roughage,GL: Green Liquid, DL: Dry Liquid, GS: Green Liquid and DS: Dry solid )
Suppl Figure 4: Phylogenetic clustering of 48 buffalo rumen metagenomes. A double hierarchical dendrogram was established through weight-pair group clustering methods based on the non-scaling Bay Curtis distance. The dendrogram shows the phylogenetic distribution of the microorganisms among the 48 metagenomes. The heat map depicts the relative percentage of each phylum of microorganisms in each sample. The heat map color represents the relative percentage of the microbial descriptions in each sample.

## Figures and Tables

**Table 1:** Summary of metagenomic data.

Green roughage: dry roughage (diets)	Average read length (bp)	Data (Mb)	Total size (Mb)
50%			
Liquid	146	549.4	1285.4
Solid	149	736	
75%			
Liquid	161	438	1127.85
Solid	149	689.85	
100%			
Liquid	180	800.69	1501.69
Solid	170	701	

Total size			3914.94

**Table 2:** Comparison of the carbohydrate active enzymes identified in buffalo rumen metagenome with those of two other metagenomes.

Enzymes	Termite gut (%)	Buffalo rumen (%)	Cow rumen (%)
Cellulases			
GH5	5.71	1.50	3.85
GH6	0.00	0.00	0.00
GH7	0.00	0.00	0.00
GH9	2.37	0.26	2.20
GH44	0.32	0.00	0.07
GH45	0.51	0.05	0.27
GH48	0.00	0.00	0.00
Total	**8.91**	**1.81**	**6.39**

Hemicellulases			
GH8	0.834	0.00	0.76
GH10	5.39	2.64	1.65
GH11	1.09	0.15	0.69
GH12	0.00	0.00	0.00
GH26	1.28	1.03	0.76
GH28	0.77	0.21	0.48
Total	**9.36**	**4.04**	**4.34**

Debranching enzymes			
GH62	0.00	0.00	0.00
GH67	1.34	2.49	0.62
GH78	0.44	2.85	3.85
Total	**1.78**	**5.34**	**4.47**

Oligosaccharide degrading enzymes			
GH1	1.86	0.05	0.21
GH2	4.36	9.85	11.68
GH3	11.94	18.3	13.87
GH29	0.89	2.28	2.06
GH35	0.38	0.78	0.62
GH38	2.63	0.26	0.89
GH39	0.44	0.00	0.21
GH42	2.76	0.10	0.27
Total	**25.28**	**31.62**	**29.81**

**Table 3:** Phylogenetic classification at domain level.

Domain	50%				75%				100%			
	Green roughage (%)		Dry roughage (%)		Green roughage (%)		Dry roughage (%)		Green roughage (%)		Dry roughage (%)	
	Liquid	Solid	Liquid	Solid	Liquid	Solid	Liquid	Solid	Liquid	Solid	Liquid	Solid
Bacteria	89.6	96.0	95.3	96.3	96.9	97.4	96.5	96.8	97.4	97.4	97.0	97.6
Archea	0.8	1.8	0.8	1.7	0.8	0.8	0.7	1.0	0.6	0.9	0.8	0.8
Eukaryota	9.1	1.9	3.4	1.8	1.7	1.5	2.3	2.0	1.7	1.6	1.7	1.4
Viruses	0.3	0.1	0.3	0.1	0.4	0.1	0.3	0.1	0.2	0.1	0.3	0.1
Unclassified	0.2	0.2	0.2	0.1	0.2	0.1	0.2	0.2	0.2	0.2	0.2	0.2
