# Supplementary material for: High Potential Source for Biomass Degradation Enzyme Discovery and Environmental Aspects Revealed through Metagenomics of Indian Buffalo Rumen
Source: Biomed Res Int. 2014 Jul 17;2014:267189. doi: 10.1155/2014/267189 (PMC4124647; doi:10.1155/2014/267189)
Supplement: Supplementary file 1 — Table S1: Assignment of Gene Fragments to Functional Groups. Table S2: Gene Assignment to Taxonomic group (48 Metagenomes). Suppl Figure1: Metabolic clustering of 48 buffalo rumen metagenomes. A double hierarchical dendrogram was established through weight-pair group clustering methods based on the non-scaling Bay Curtis distance. The dendrogram shows the functional categories of the 48 metagenomes. The linkages of the dendrogram are based on the relative abundance of metabolic profiles. The heat map depicts the relative percentage of each phylum of microorganisms in each sample. The heat map color represents the relative percentage of the microbial descriptions in each sample. Suppl Figure 2: A comparative abundance of organism with variable roughage diet respect to 50%, 75% and 100% GR and DR in GL, DL, GS and DS phase at Phyla level. (GR: Green roughage, DR: Dry roughage, GL: Green Liquid, DL: Dry Liquid, GS: Green Liquid and DS: Dry solid) Suppl Figure 3: A comparative abundance of organism with variable roughage diet respect to 50%, 75% and 100% GR and DR in GL, DL, GS and DS phase at Genus level. (GR: Green roughage, DR: Dry roughage,GL: Green Liquid, DL: Dry Liquid, GS: Green Liquid and DS: Dry solid ) Suppl Figure 4: Phylogenetic clustering of 48 buffalo rumen metagenomes. A double hierarchical dendrogram was established through weight-pair group clustering methods based on the non-scaling Bay Curtis distance. The dendrogram shows the phylogenetic distribution of the microorganisms among the 48 metagenomes. The heat map depicts the relative percentage of each phylum of microorganisms in each sample. The heat map color represents the relative percentage of the microbial descriptions in each sample. [file 267189.f1.zip › Supp.Figure_2.docx]

Figure 2 Supp 2- A comparative abundance of organism with variable roughage diet respect to 50%, 75% and 100% GR and DR in GL, DL, GS and DS phase at Phyla level.

(GR: Green roughage, DR: Dry roughage, GL: Green Liquid, DL: Dry Liquid, GS: Green Liquid and DS: Dry solid)
